# Anti-Inflammatory Activity of Piquerol Isolated from *Piqueria trinervia* Cav.

**DOI:** 10.3390/ph15070771

**Published:** 2022-06-21

**Authors:** Nimsi Campos-Xolalpa, Ana Laura Esquivel-Campos, Rubria Marlen Martínez-Casares, Salud Pérez-Gutiérrez, Julia Pérez-Ramos, Ernesto Sánchez-Mendoza

**Affiliations:** Departamento de Sistemas Biológicos, Universidad Autónoma Metropolitana-Xochimilco, Calzada del Hueso 1100, Ciudad de México 04960, Mexico; nimsicaxo@hotmail.com (N.C.-X.); aesquivel@correo.xoc.uam.mx (A.L.E.-C.); rmartinezc@correo.xoc.uam.mx (R.M.M.-C.); msperez@correo.xoc.uam.mx (S.P.-G.)

**Keywords:** cytokines, macrophages, monoterpene

## Abstract

Background: Inflammation is a complex process as a response to several stimuli, such as infection, a chemical irritant, and the attack of a foreign body. Piquerol was isolated from *Piqueria trinervia*, and its anti-inflammatory activity was evaluated using in vivo and in vitro models. Methods: Piquerol is a monoterpene that was identified using NMR, FT-IR spectroscopy, and mass spectrometry analysis. The anti-inflammatory activity was tested in vivo in ear edema induced with TPA in mice. Piquerol was also tested on J774A.1 macrophages stimulated with lipopolysaccharide (LPS), and the levels of NO, NF-κB, TNF-α, IL-1β, IL-6, and IL-10 were determined using ELISA. Results: The results show that piquerol diminished ear edema (66.19%). At 150.51 µM, it also inhibited the levels of NO (31.7%), TNF-α (49.8%), IL-1β (69.9%), IL-6 (47.5%), and NF-κB (26.7%), and increased the production of IL-10 (62.3%). Piquerol has a membrane stabilization property in erythrocyte, and at 100 µg/mL, the membrane protection was of 86.17%. Conclusions: Piquerol has anti-inflammatory activity, and its possible mechanism of action is through the inhibition of pro-inflammatory mediators. This compound could be a candidate in the development of new drugs to treat inflammatory problems.

## 1. Introduction

Inflammation is a complex process as a response to several stimuli, such as infection, a chemical irritant, and the attack of a foreign body [1]. During the inflammatory process, exogenous and endogenous mediators are released, with the main objective of tissue repair and the restoration of homeostasis. Macrophages are cells that are highly responsive to the pathological inflammatory process and are responsible for the synthesis and release of many mediators; the activation of nuclear kappa (NF-κB) drives the expression of chemokines and pro-inflammatory cytokines [2], including tumor necrosis factor (TNF-α), interleukin-1β (IL-1β), and IL-6 [3]. NF-κB is also involved in other processes, such as apoptosis and tumorigenesis [2].

Non-steroidal anti-inflammatory drugs (AINES) were discovered more than 100 years ago and remain at the top of the list for the pharmacological management of inflammatory diseases. However, they are commonly associated with a high incidence of adverse effects, particularly in the gastrointestinal tract and cardiovascular system [4]. Therefore, there is growing interest in identifying new drugs with greater therapeutic efficacy and reduced toxic effects. Within the broad range of compounds obtained naturally, terpenes have been gaining importance, representing the great majority of components identified in essential oils, with monoterpenes and sesquiterpenes prevailing, and being found in many plants in nature [5]. Some of these compounds have been shown to have pharmacological activity, and studies have shown that different monoterpenes, such as m-cymene and L-carveol, have anti-inflammatory activity in vitro by the suppression of NO and NF-κB signaling pathways [6,7].

*Piqueria trinervia* Cav., known as “hierba de San Nicolas”, belongs to the Asteraceae family and is an herbaceous plant that grows in different regions of Mexico [8], used in traditional medicine to relieve pain, to treat malaria, and as an antipyretic. Piquerol A, piquerol B, trinervinol, and carquejilo acetate have been isolated from *P. trinervia* [9]. Piquerol has been tested as a molluscicidal agent against eight species of pulmonated snails [10], evaluated for its inhibitory effect on the growth of epimastigotes of *Trypanosoma cruzi* [11], and shown to have antimicrobial effects against pathogenic bacteria [12]. In 2017, Rufino-González et al. found that piquerol and trinervinol had anti-giardia effects against *Giardia intestinalis* [13]; however, there are no reports about the anti-inflammatory activity of *P. trinervia*. Therefore, in this study, we isolated piquerol from *P. trinervia* and evaluated its anti-inflammatory activity in an in vivo model and in vitro in macrophages stimulated with lipopolysaccharide (LPS).

## 2. Results

### 2.1. Structure of Piquerol

**Figure 1:**
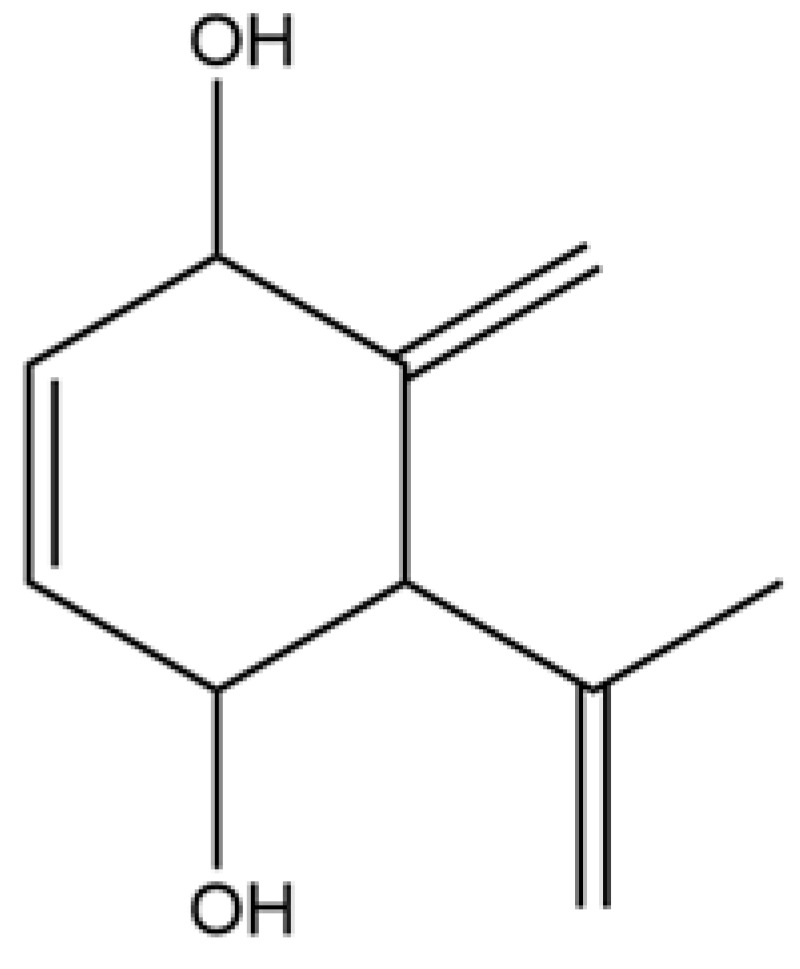
Piquerol chemical structure.

White crystals were isolated from the dichloromethane extract in the 65:35 fraction of a hexane–ethyl acetate mixture. Spectroscopic and spectrometric analysis confirmed this compound as piquerol (Figure 1 and Figures S1–S4, Supplementary Materials), which is a monoterpene previously reported by Romo et al. [9], and its crystallographic data were reported by Soriano-García et al. [14]. In this study, the spectroscopic and spectrometric data are as follows: ^1^H NMR (600 MHz, chloroform-d) δ 6.03 (ddd, J = 9.7, 4.7, 2.0 Hz), 5.86 (dd, J = 9.7, 2.9 Hz), 5.45–5.41 (m), 5.36 (dt, J = 2.2, 1.1 Hz), 5.09 (t, J = 1.7 Hz), 5.03 (q, J = 1.8 Hz), 4.66–4.58 (m), 4.34 (ddd, J = 10.4, 4.7, 3.2 Hz), 3.33 (dd, J = 8.7, 2.9 Hz), 2.94 (s), 2.12 (d, J = 10.4 Hz), 1.79 (s); ^13^C NMR (151 MHz, chloroform-d) δ 146.09, 142.70, 132.99, 131.98, 113.86, 110.28, 77.21, 70.34, 67.97, 52.27, 23.45; FT-IR, 3263.8 cm^−1^ (OH), 3177 cm^−1^ (=C-H), 2920, 2850.7, 2826.17 cm^−1^, and 1370 (C-H), 1661 and 1642 cm^−1^ (C=C), 1083 cm^−1^ (C-O); [α]^25^_D_ = +96.94 (MeOH); CG, t_R_ = 9.9 min and 166.10 *m*/*z*.

### 2.2. Anti-Inflammation Activity In Vivo

Table 1 shows the differences in the weight of the ears of each evaluated group. The negative group is significantly different (*p* < 0.05) compared to the IND and piquerol groups, and also, between the IND and piquerol groups, there is no significant difference (*p* > 0.05). Therefore, piquerol at doses of 2 mg/ear diminished ear edema induced with TPA in mice by 66.19%, which was similar to the effect obtained with the group treated with indomethacin (IND), which inhibited 61.44%.

### 2.3. Measurement of Mediators: Pro-Inflammatory and Anti-Inflammatory

The cell viability of piquerol was evaluated on J774A.1 macrophages at concentrations of 37.62 to 1204 μM. The cytotoxic activity of piquerol showed the IC_50_ = 1151.71 µM; therefore, a concentration of 150.51 μM was used in the next experiments.

**Figure 2:**
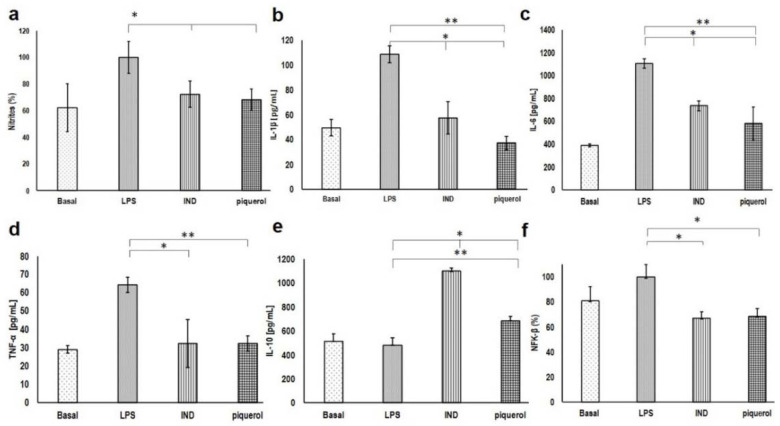
Activity of piquerol and IND at 150.51 μM and 47.8 μM, respectively, in J774A.1 macrophages stimulated with LPS on the production of (**a**) NO; (**b**) IL-1β; (**c**) IL-6; (**d**) TNF-α; (**e**) IL-10; and (**f**) NF-κB. The graph represents the mean ± standard deviation of three independent experiments (*n* = 6). ** *p* < 0.01 and * *p* < 0.05 statistically significant difference compared (**a**–**d**,**f**) with LPS, and IL-10 (**e**) piquerol group was compared to IND group.

J774A.1 macrophages were stimulated with LPS to increase the production of NF-κB, NO, IL-1β, IL-6, and TNF-α. The stimulated macrophages were treated with piquerol and IND at concentrations of 150.51 μM and 47.8 μM, respectively, and the production of NO was shown to decrease by 31.7% and 28.0%, respectively. The inhibitory effect of piquerol was similar to that observed with IND (Figure 2a).

Piquerol also diminished other mediators involved in the inflammatory process, such as the cytokines IL-1β, IL-6, and TNF-α. The level of IL-1β decreased in the groups treated with piquerol and IND by 65.9% and 47.3%, respectively (Figure 2b), while IL-6 production also reduced by 47.5% and 33.3%, respectively (Figure 2c). Piquerol showed a very significant decrease (*p* < 0.01) compared with LPS, and the concentration of TNF-α also diminished by 50.2% and 50.2%, respectively (Figure 2d); that is, the effect of piquerol was similar to that obtained with IND, without statistical difference.

IL-10 is an anti-inflammatory cytokine that is produced in order to re-establish homeostasis. Piquerol increased the concentration of this interleukin 63.6% (Figure 2e). 

NF-κB is an inducible transcription factor that can activate the transcription of various genes and thereby regulate inflammation. NF-κB increases the production of inflammatory cytokines, chemokines, and adhesion molecules. Piquerol inhibited the production of NF-κB in J774A.1 macrophages stimulated with LPS by 26.6% (Figure 2f), which was similar to the effect seen with IND (25.0%). 

### 2.4. Membrane Stabilization Property

Piquerol at concentrations of 25, 50, 100, and 200 μg/mL significantly inhibited the hypotonic-solution-induced lysis of the human red cell membrane. At these concentrations, piquerol showed the percentage of protection on the membrane stabilization of erythrocytes of 86.98%, 85.20%, 86.17%, and 76.65%, respectively, (Table 2), which was comparable to that obtained with diclofenac (87.93%, 84.19%, 84.97%, and 77.84%, respectively). These results indicate that piquerol promotes the reduction in inflammation since it protects the erythrocyte membrane in a similar way to that of diclofenac.

## 3. Discussion

Many monoterpenes have different biological activities, such as cytotoxic, anti-parasitic, anti-microbial, and anti-inflammatory activities, among others [11,15,16,17]. In this study, we determined for the first time that piquerol, a monoterpene isolated from *P. trinervia*, has anti-inflammatory activity.

In auricular edema induced by TPA in mice, the TPA promotes inflammation by activating protein kinase C, prostaglandins, and phospholipase A2, NF-κB, IL-1β, and TNF-α, among others [18,19]. The administration of piquerol significantly reduced the inflammation, and its activity was similar to the reference drug (IND).

During inflammation, the lysosome membrane ruptures, releasing a variety of enzymes, with the discharge of lysosomal contents resulting in acute inflammation and connective tissue degradation [20]. NSAIDs inhibit the release of lysosomal enzymes or stabilize the lysosomal membranes [21]. Furthermore, the compounds that have an effect on the membrane stabilization can inhibit the production of phospholipases by diminishing the release of different inflammatory mediators [22]. Piquerol inhibited the induced lysis of erythrocyte membranes, which are considered similar to lysosomal membranes. These results suggest that the anti-inflammatory effect of piquerol might be associated with phospholipase release.

The inflammation of mediators, such as NO, TNF-α, IL-1β, and IL-6, among others, is activated due to the presence of tissue damage or infection; therefore, the concentrations of these mediators increase in the damaged areas [6]. LPS promotes an acute inflammatory process in macrophages through the production of these inflammatory mediators [23]. Additionally, Tong et al. demonstrated that the inflammation induced by LPS in macrophages promotes the phosphorylation of JNK, ERK, and p38 MAPK. Furthermore, it is well established that MAPK activation is involved in the production of inflammatory mediators via LPS-stimulated macrophages [24].

The overproduction of NO induces inflammation in abnormal conditions [25]; this compound promotes the release of cytokines, chemokines, and endothelial–leukocyte adhesion molecules, inducing vasodilation and the production of reactive nitrogen species, which are involved in tissue damage [26].

IL-1β is an interleukin that plays an important function in normal conditions, such as sleep, temperature, and the regulation of feeding; however, this cytokine is implicated in host-defense responses against different disease conditions, such as infections, arthritis, and osteoarthritis [27], increasing tissue damage [28]. IL-1β is also involved in pain progression [29].

IL-6 issues a warning signal as a result of a damage event, such as a tissue injury or an infection [30], contributing to the host defense by stimulating acute phase responses; however, IL-6 has a pathological effect of chronic inflammation [31].

TNF-α is a pro-inflammatory cytokine that plays an important role in innate and adaptive immunity [32] and regulates the release of other pro-inflammatory cytokines, such as IL-6. This cytokine plays an important role in vasodilatation and the formation of edema [33].

IL-10 is an interleukin with a potent anti-inflammatory effect. It has an important role in the prevention of autoimmune and inflammatory pathologies, thus maintaining health [34]. Therefore, IL-10 production helps regulate the inflammatory process, and this cytokine promotes the diminution of mediators NF-κB, NO, TNF-α, IL-1β, and IL-6, consequently reducing inflammation.

NF-κB is a transcription factor involved in inducing the expression of different pro-inflammatory cytokines and has an important role in the activation and differentiation of inflammatory T cells. The inhibition of NF-κB therefore contributes to the treatment of various inflammatory diseases [35].

In this study, we found that piquerol diminished the release of NO, TNF-α, IL-1β, IL-6, and NF-κB and promoted IL-10 production in macrophages stimulated with LPS in the same proportion as the reference drug. The reduction in these pro-inflammatory mediators and promoters suggests that the MAPK pathway is being affected.

In summary, piquerol downregulated the production of NF-κB and the pro-inflammatory cytokines, IL-1β, TNF-α, and IL-6, and significantly increased the release of anti-inflammatory cytokine IL-10. The results obtained in this study suggest that the anti-inflammatory effect of piquerol must be due to the inhibition of the protein NF-κB, which promotes the production of pro-inflammatory mediators and protein kinase C. 

The inhibition of NO, TNF-α, IL-1β, IL-6, and NF-κB and the increase in IL-10 are the targets for the treatment of inflammation. The demonstrated anti-inflammatory activity of piquerol supports the use of this compound as an alternative for the resolution of inflammation associated with different chronic degenerative diseases, particularly taking into account the fact that piquerol shows low toxicity in J774A.1 macrophages.

## 4. Materials and Methods

### 4.1. Material

Dulbecco’s Modified Eagle’s Medium (DMEM), fetal bovine serum (FBS), antibiotics, 3-(4,5-dimethylthiazol-2-yl)-2,5-diphenyl tetrazolium bromide (MTT), indomethacin (IND), dimethyl sulfoxide (DMSO), polyvinylpyrrolidone (PVP), Griess reagent, 12-O-tetradecanoylphorbol-13-acetate (TPA), and lipopolysaccharide (LPS) were purchased from Sigma Aldrich (St. Louis, MO, USA). The murine J774A.1 macrophages were purchased from ATCC (Manassas, VA, USA). The enzyme-linked immunosorbent assay (ELISA) kits were from TONBO bioscience.

### 4.2. Plant Material

The aerial parts of *Piqueria trinervia* Cav. were collected in August 2019 from highway 70 SLP-Rio Verde (22.0942829; −100.7173475). The plant was identified by M. en C. Gabriel Flores Franco of the Universidad Autónoma del Estado de Morelos (UAEM), and a voucher specimen (HUMO37542) was placed in the HUMO herbarium of the UAEM. 

### 4.3. Extraction and Isolation of Piquerol

The aerial parts of *P. trinervia* were shade dried and ground. The extract was prepared by maceration with 300 g of vegetal material and 2 L of dichloromethane (CH_2_Cl_2_) for 8 days. The mixture was filtered, CH_2_Cl_2_ was eliminated under reduced pressure, and the extract was separated using open column chromatography packed with silica gel (Macherey-Nagel 60, 70–230 mesh ASTM). The mobile phase was a hexane–ethyl acetate mixture, and the polarity was increased. A white crystalline compound was obtained (yield 0.014%) with an m.p. of 138–139 °C in the 65:35 fraction (hexane–ethyl acetate). The purity of the compound was determined using thin-layer chromatography, and the structure was elucidated using NMR, IR, and mass spectrometry.

### 4.4. Structural Analysis

Infrared spectra were obtained using a Spectrum Two FT-IR spectrometer (Perkin Elmer, Waltham, MA, USA) with Attenuated Total Reflection (ATR); the samples were analyzed by forming a film with chloroform. NMR spectra were recorded in a CDCl_3_ solution at 299 K on an Agilent DD2 600 spectrometer. The ^1^H and ^13^C NMR chemical shifts were reported relative to TMS and CDCl_3_, respectively. The GC-MS spectral data were digitalized using the Mass Spectrum Digitizer program from the National Institute of Standards and Technology (NIST). The optical rotation of the compound was determined using a Perkin Elmer polarimeter 341 in methanol (MeOH).

### 4.5. Animals

Male CD1 mice (20–25 g) were from the Universidad Autónoma Metropolitana-Xochimilco animal facility. The experimental protocol (No. 140) was approved by the Research Bioethics Committee of the Universidad Autónoma Metropolitana-Xochimilco. Mice were maintained with free access to food and water and were housed at 24 °C ± 1 °C with 12 h light/dark cycles.

### 4.6. Acute Anti-Inflammatory Activity: Edema Induced by TPA

Groups of eight CD1 mice were topically administered with a solution of TPA (2.5 µg/mouse) in acetone (20 µL) on the external and internal surface of the right ear of each animal, and acetone (vehicle) was administered to the internal surface and outer left ear. After 30 min, 2 mg/ear of IND (reference drug group), 2 mg/ear of piquerol (test group), or 20 µL/vehicle (negative control group) was administered to the right ears of the mice, while vehicle was applied to the left ear. After 6 h, the animals were sacrificed, and 6 mm portions of the central sections of both ears were obtained. The portions were weighed to obtain the percentage of edema inhibition using the following equation 2 [36]:

(1) $$\%\mathrm{Inhibition}=\left(\;\frac{\left(\mathrm{Wt}-\mathrm{Wnt}\right)\;\mathrm{control}-\left(\mathrm{Wt}-\mathrm{Wnt}\right)\;\mathrm{treated}}{\left(\mathrm{Wt}-\mathrm{Wnt}\right)\;\mathrm{control}}\right)\times 100$$

Wt: weight of treated ear, Wnt: weight of non-treated ear.

### 4.7. Cell Viability Assay

The viability of the macrophages treated with piquerol was determined as follows: 5000 cells/well were seeded in a 96-well plate; piquerol was applied at concentrations of 37.6 to 1204 μM for 24 h; 10 μL of MTT (5 mg/mL) solution was then placed in each well; 4 h later, the medium was eliminated, and the formazan crystals were dissolved with DMSO. The absorbance was determined at 540 nm [37]. The IC_50_ was calculated using linear regression.

### 4.8. Determination of Nitric Oxide (NO), Cytokines, and NF-κB Levels

Initially, 5 × 10^5^ J774A.1 macrophages were seeded per well in 6-well plates, and the cells were stimulated with LPS (5 μg/mL). After 2 h, the macrophages were treated with 150.51 μM piquerol, IND reference drug (47.8 µM), or vehicle. After 24 h, the supernatants were obtained for the quantification of IL-1β, IL-6, IL-10, and TNF-α, as well as NF-κB (in cytoplasm), using commercial ELISA, following the manufacturer’s instructions. The absorbance was determined at 405 nm.

For the quantitation of NO production, 100 μL of supernatant and 100 μL of Griess reagent was placed in a 96-well plate, the plate was incubated at 37 °C for 30 min, and the absorbance was determined at 540 nm [38]. A nitric oxide production of 100% was considered for the LPS group.

### 4.9. Methodology of Membrane Stabilization Property

Blood samples were collected in aseptic conditions from healthy human volunteers who did not consume NSAIDS, steroids, or oral contraceptives for two weeks. Blood samples were washed with an equal volume of Alsever solution (2% dextrose, 0.8% sodium citrate, 0.05% citric acid, and 0.42% sodium chloride in water), centrifuged at 3000 rpm for 10 min, and packed cells were washed three times with Alsever solution.

A suspension of 5% erythrocytes was mixed with different concentrations (25–200 μg/mL) of piquerol or diclofenac prepared in PBS buffer. Distilled water and PBS buffer were used as negative controls. All samples were incubated at 37 °C for 30 min and centrifuged at 3500 rpm for 5 min [39]. Optical density was read at 450 nm. The %Protection was calculated with Equation (2):

(2) $$\%\mathrm{Protection}=100-\left(\frac{\mathrm{optical}\;\mathrm{density}\;\mathrm{of}\;\mathrm{Test}\;\mathrm{sample}}{\mathrm{Optical}\;\mathrm{density}\;\mathrm{of}\;\mathrm{Control}}\times 100\right)$$

### 4.10. Statistical Analysis

The results are expressed as the mean ± SD. Statistical analyses were performed with an ANOVA test and Tukey’s post hoc test, using the statistical program iner-STAT20-a v1.3. Values of * *p* < 0.05 and ** *p* < 0.01 were considered statistically significant.

## 5. Conclusions

Plants are an important source of compounds with different biological activities. Piquerol, isolated from *Piqueria trinervia* Cav., showed anti-inflammatory activity in vitro and in vivo models, its possible mechanism of action is through the inhibition of NO, and pro-inflammatory cytokine levels (IL-1β and IL-6), as well as the increase of the levels of the anti-inflammatory cytokine IL-10. This monoterpene reduced the acute inflammation induced by TPA in the mouse ear, also is a protective agent against membrane lysis. The results suggest that Piquerol might be used in the treatment of inflammatory conditions.

## Figures and Tables

**Table 1:** Acute anti-inflammatory activity: edema induced by TPA.

Group	Difference of Weight (mg)	% Decrease in Inflammation
Negative	11.45 ± 0.5 **	0.0
IND (2 mg/ear)	4.41 ± 0.5 *	61.44 ± 4.2
Piquerol (2 mg/ear)	4.36 ± 0.6 *	66.19 ± 5.3

The values are the mean ± S.E.M. (*n* = 8). * *p* < 0.05 statistically significant difference compared with negative group and ** *p* < 0.05 statistically significant difference compared with IND group.

**Table 2:** Percent human red blood cell membrane stabilization of piquerol.

µg/mL	Diclofenac	Piquerol
200	77.84 ± 0.32	76.65 ± 1.21
100	84.97 ± 0.45	86.17 ± 0.47
50	84.19 ± 0.11	85.20 ± 1.22
25	87.93 ± 0.56	86.98 ± 0.14

The values are the mean ± S.E.M. of three independent experiments (*n* = 4).

## Data Availability

Data is contained within the article and Supplementary Materials.

## Supplementary Materials

The following supporting information can be downloaded at: https://www.mdpi.com/article/10.3390/ph15070771/s1, Figure S1: FT-IR spectrum; Figure S2: GC/MS chromatogram (a) and MS spectrum (b); Figure S3: ^1^H-NMR spectrum; Figure S4: ^13^C-NMR spectrum.
