# Supplementary material for: Anti-Inflammatory Activity of Piquerol Isolated from Piqueria trinervia Cav
Source: Pharmaceuticals (Basel). 2022 Jun 21;15(7):771. doi: 10.3390/ph15070771 (PMC9324079; doi:10.3390/ph15070771)
Supplement: Supplementary file 1 [file pharmaceuticals-15-00771-s001.zip › pharmaceuticals-1765397-supplementary.pdf]

pharmaceuticals

Article

Anti-inflammatory activity of piquerol isolated from *Piqueria trinervia* Cav.

Nimsi Campos-Xolalpa, Ana Laura Esquivel-Campos, Rubria Marlen Martínez-Casares, Salud Pérez-Gutiérrez, Julia Pérez-Ramos * and Ernesto Sánchez-Mendoza *

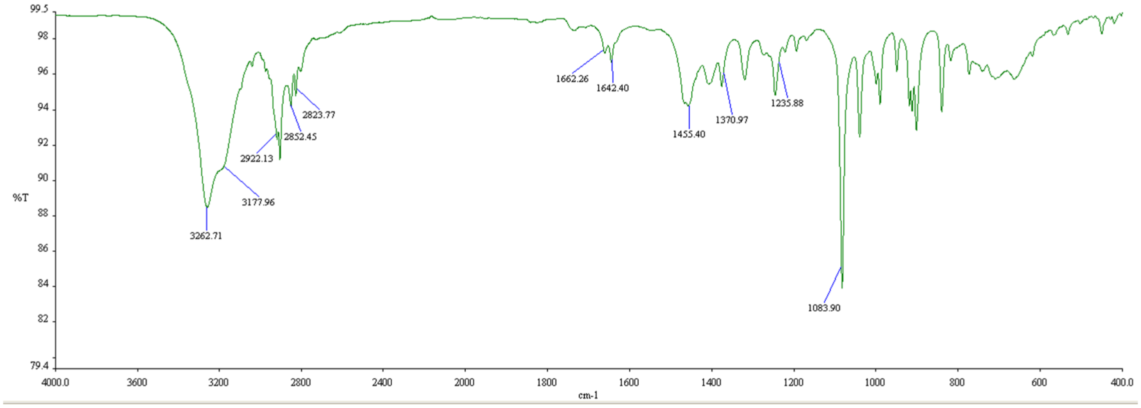

Figure S1. Spectrum of TF-IR of Piquerol

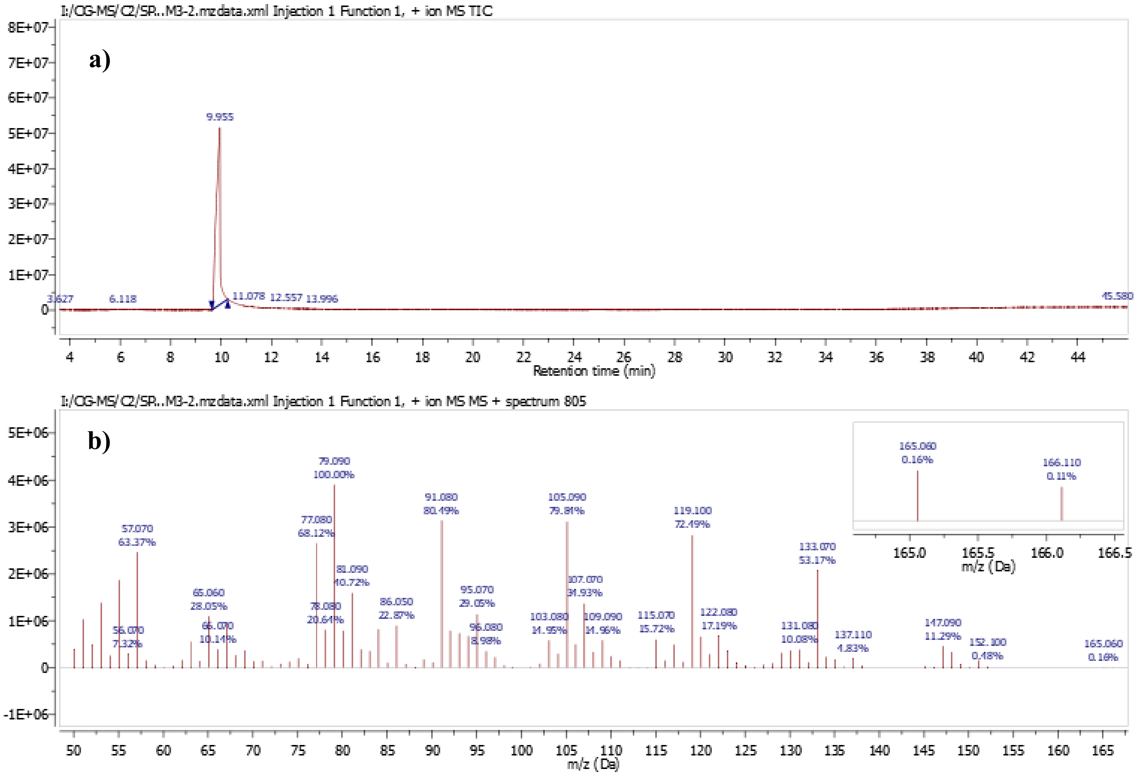

Figure S2. GC/MS chromatogram (**a**) and spectrum (**b**) of the Piquerol of *Piqueria trinervia*. The peak at 9.95 min corresponds to piquerol con 166.11 m/z

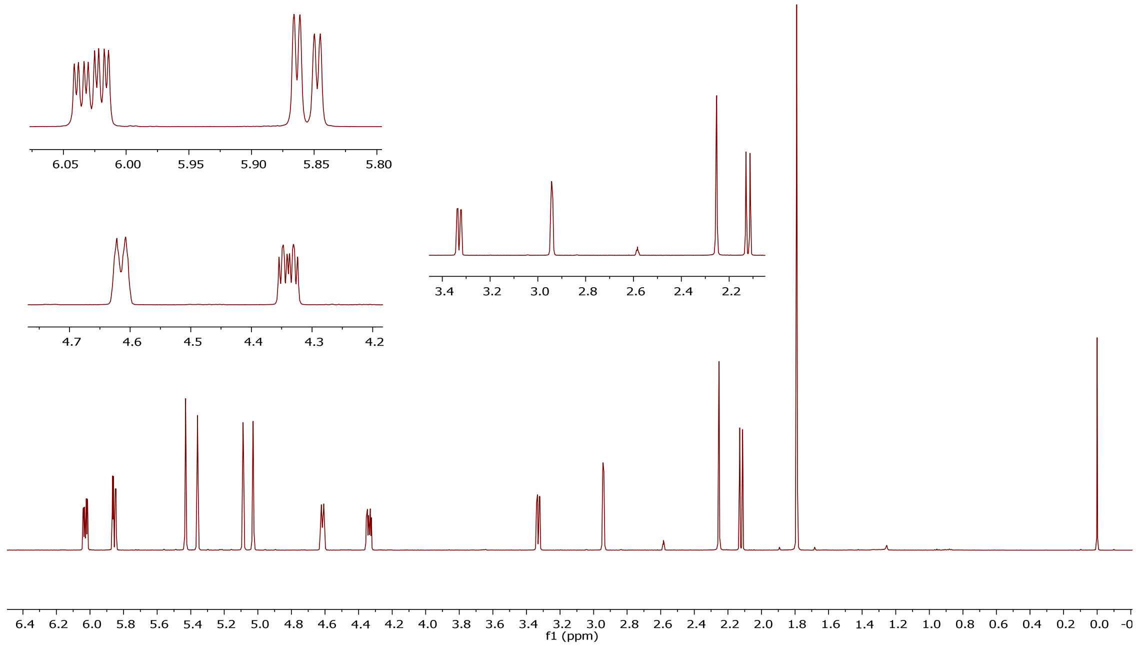

Figure S3. Piquerol ^{1}H-NMR spectrum.

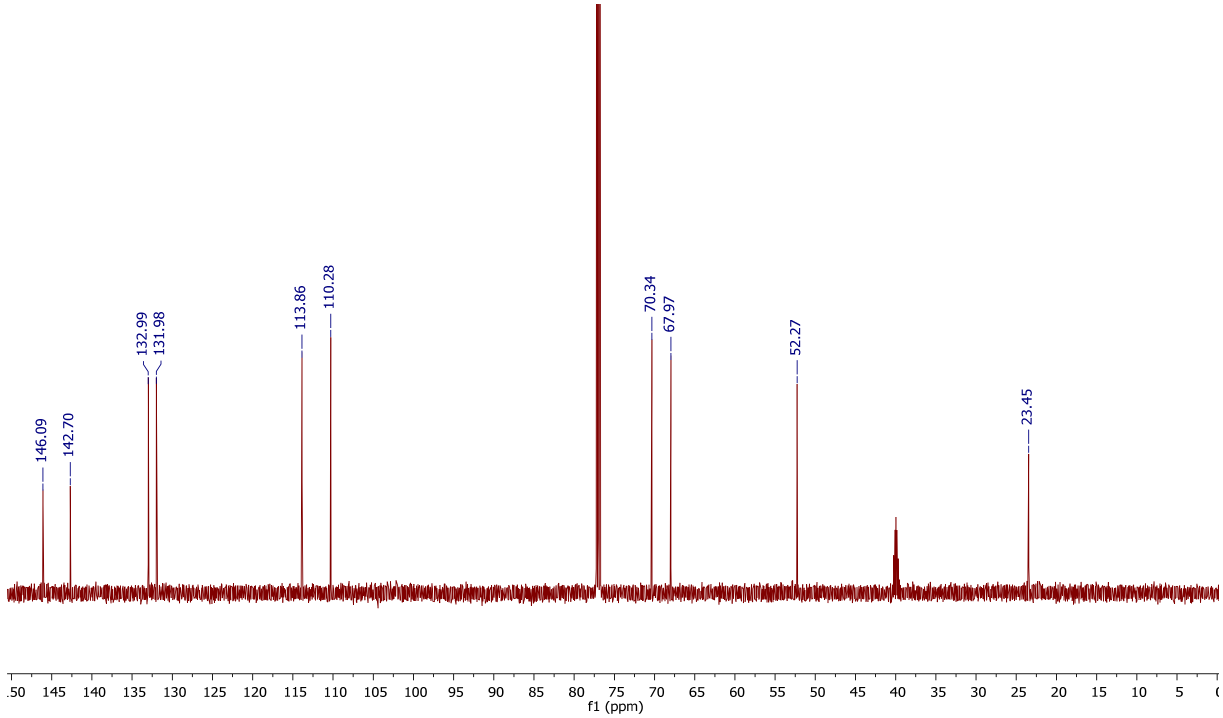

Figure S4. Piquerol ^{13}C-NMR spectrum.
